# Determinants and Prognostic Significance of Symptomatic Status in Patients with Moderately Dysfunctional Bicuspid Aortic Valves

**DOI:** 10.1371/journal.pone.0169285

**Published:** 2017-01-06

**Authors:** Soo Youn Lee, Chi Young Shim, Geu-Ru Hong, In Jeong Cho, Hyuk-Jae Chang, Jong-Won Ha, Namsik Chung

**Affiliations:** Department of Cardiology, Severance Cardiovascular Hospital, Yonsei University College of Medicine, Seoul, Korea; Harvard Medical School, UNITED STATES

## Abstract

**Background:**

We aimed to identify the clinical and echocardiographic determinants of symptoms and their prognostic implications in patients with moderately dysfunctional bicuspid aortic valves (BAVs).

**Methods:**

Among 1,019 subjects in the BAV registry treated in a single tertiary care center, the records of 127 patients (85 men, age 58±13 years) with moderately dysfunctional BAVs were comprehensively reviewed. The patients were divided into two groups based on symptom status: asymptomatic (n = 80) vs. symptomatic (n = 47). The primary end-point was defined as a composite of aortic valve surgery, hospitalization for heart failure, and any cause of death.

**Results:**

The symptomatic group had a higher proportion of females, hypertension, aortic stenosis, and aortopathy than did the asymptomatic group. The symptomatic group showed lower e′ (5.5±1.7 vs. 6.5±2.2 cm/s, p = 0.003), higher E/e′ (13.3 ± 4.9 vs. 10.9±3.7, p = 0.002), and larger left atrial volume index (29.9±11.4 vs. 24.6±9.1 ml/m^2^, p = 0.006) than did the asymptomatic group. In multivariate logistic regression analysis, female gender (odds ratio [OR] 2.84, 95% confidence interval [CI] 1.10–7.36, p = 0.031), hypertension (OR 3.07, 95% CI 1.20–7.82, p = 0.019), moderate aortic stenosis (OR 5.33 5.78, 95% CI 1.99–16.83, p = 0.001), E/e′ >15 (OR 3.82, 95% CI 1.03–11.19, p = 0.015), and aortopathy (OR 2.76, 95% CI 1.07–7.10, p = 0.035) were independently correlated with symptom status. The symptomatic group showed a significantly lower rate of event-free survival during the 8-year follow-up period (54±9% vs. 68±10%, p = 0.001).

**Conclusions:**

In patients with moderately dysfunctional BAVs, the presence of moderate aortic stenosis, aortopathy, and diastolic dysfunction determines symptom status, along with female gender and hypertension. Symptom status was associated with clinical outcomes.

## Introduction

Bicuspid aortic valve (BAV) is known to frequently progress to aortic valve stenosis (AS) and aortic regurgitation, requiring aortic valve replacement (AVR). Furthermore, dilatation of the aortic root and/or the ascending aorta (AA) occurs more frequently in patients with a bicuspid aortic valve (BAV) than in patients with a tricuspid aortic valve (TAV). Recent studies have demonstrated that left ventricular (LV) diastolic function is impaired in subjects with a normally functioning BAV as compared to subjects with a TAV, and LV diastolic dysfunction is associated with aortic dilatation and consequent aortic stiffness [1–4]. However, the clinical implications of diastolic dysfunction in BAV subjects with aortopathy are uncertain.

In patients with moderate to severe aortic stenosis (AS) or aortic regurgitation (AR), LV hypertrophy and diastolic dysfunction are quite commonly caused by chronic pressure or volume overload and LV hypertrophy is related to impaired relaxation and increased LV chamber stiffness [5,6].

The occurrence of symptoms and adverse events are most likely related to the global hemodynamic burden faced by the ventricle [7]. This global load not only includes the valvular load, but also the pulsatile and steady components of arterial load, which are related to reduced arterial compliance and increased vascular resistance [7]. Recently, the clinical importance of diastolic dysfunction in patients with severe AS has been highlighted with regards to understanding symptom status and predicting clinical outcomes [8,9]. Moreover, the importance of valvular, arterial, and ventricular interplay in AV disease has been suggested for improving risk stratification and identifying patients who could benefit from early elective aortic valve surgery [10]. Increased aortic stiffness is independently associated with elevated LV filling pressure, plasma brain natriuretic peptide level, and symptom severity in AS [11].

Therefore, we hypothesised that 1) the presence of symptoms is determined by BAV phenotype or function, aortic phenotype, or LV diastolic function in patients with moderately dysfunctional BAVs, and 2) symptomatic patients show worse clinical outcomes than do asymptomatic patients. In order to test our hypotheses, we reviewed clinical and echocardiographic characteristics and clinical events in patients with moderately dysfunctional BAVs.

## Materials and Methods

### Patient population

**Fig 1:**
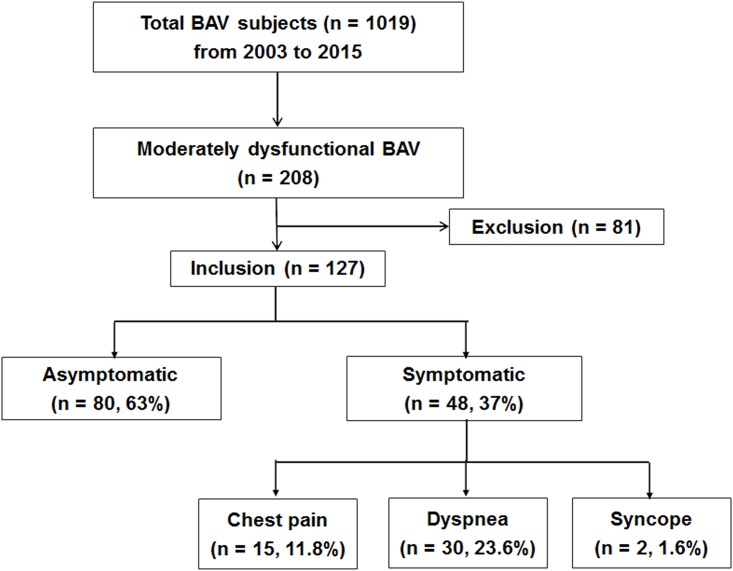
Description of the study population.

We retrospectively reviewed the echocardiographic database and medical records of patients with BAVs who were diagnosed from 2003 to 2015 at Severance Cardiovascular Hospital (Yonsei University College of Medicine, Seoul, Republic of Korea). During this period, a total of 1,019 patients with BAVs were identified. Among them, 208 patients who had moderate AS and/or AR evident on a transthoracic echocardiogram defined using the guidelines in place [12,13] at the time of diagnosis were included in our study. All patients diagnosed with severe AS and/or AR by echocardiogram were excluded. Patients who had coronary artery disease, defined as >50% narrowing in at least one coronary artery on an angiogram (n = 38), an LV ejection fraction <50% (n = 18), previous open heart surgery (n = 4), concomitant other valvular diseases of moderate or severe status (n = 6), infective endocarditis (n = 6), hypertrophic cardiomyopathy (n = 4), end-stage renal disease (n = 4), and chronic obstructive lung disease (n = 1) were excluded. Therefore, 127 patients (mean age 58±13 years, 85 men) were ultimately included in this study. All patients’ medical records, which were recorded by physicians, were carefully reviewed by a single cardiologist. History of cardiac symptoms, including dyspnoea according to New York Heart Association classes, angina, syncope, or presyncope, were reported at the time of the initial clinical and echocardiographic evaluation. Based on their presenting symptoms, including chest pain, dyspnoea, or syncope, the study population was divided into two groups: asymptomatic (n = 80, 63%) vs. symptomatic (n = 47, 37%) (Fig 1). The Institutional Review Board of Yonsei University College of Medicine approved the present study, which was conducted in compliance with the Declaration of Helsinki.

### Echocardiographic assessments

Standard two-dimensional and Doppler measurements were performed following American Society of Echocardiography guidelines [14]. A congenital BAV was diagnosed when only two cusps were unequivocally identified in systole and diastole in the short-axis view, with a clear ‘‘fish mouth” appearance during systole, as previously described [3]. We classified two BAV phenotypes based on the orientation of the free edge of the cusp, defined as the anterior-posterior and right-left forms of BAV (BAV-AP and BAV-RL, respectively) [15]. The severity of AS or AR were assessed using an integrated approach [16,17]. All measurements of the aorta were performed according to the recommendations on the QRS complex of the electrocardiogram [18]. The dimensions of the Valsalva sinuses were measured perpendicular to the right and left (or non-) aortic sinuses. The sinotubular junction was measured where the aortic sinuses met the tubular aorta. The AA was measured approximately 2 cm distal to the sinotubular junction, as described previously [3]. Aortopathy was defined as a predominant dilatation of the Valsalva sinuses or AA. Three aortic phenotypes were identified: (1) normal shape (Valsalva sinuses <39 mm and AA <Valsalva); (2) predominant dilatation of the Valsalva sinuses (Valsalva ≥39 mm and Valsalva>AA); and (3) predominant dilatation of the AA (AA ≥39 mm and AA >Valsalva). End-systolic pressure was estimated as systolic blood pressure ×0.9, as described previously [19–21]. The effective arterial elastance (Ea), a global marker of arterial stiffness that encompasses both steady and pulsatile arterial load, was calculated as the end-systolic pressure divided by the stroke volume [19–21]. To assess global LV afterload in AS patients, valvulo-arterial impedance (Zva) was calculated using a previously validated method [22]. Echocardiographic data were gathered and analyzed by experienced echocardiographers who were unaware of each patient’s clinical data.

### Follow-up

Follow-up information was obtained via a review of the medical records. The primary end-point was a composite of death, hospitalization for heart failure, and AV replacement. The clinical management of the patients was determined independently by their personal cardiologists.

### Statistical analysis

Data are presented as mean ± standard deviation or percentage unless otherwise specified. Differences between groups were compared using the Student’s t-test, and categorical variables were tested by Fisher’s exact test or Pearson’s chi-square test. In order to determine independent correlates of e′ velocity and aortic mechanical and functional properties, linear relations were verified using a simple linear regression analysis. Multiple logistic regression analysis was performed to assess the independent determinants for the presence of symptoms. All variables with suspected clinical relevance were entered, and variables were adjusted for age, gender, body mass index, and a history of hypertension or diabetes mellitus. Their incremental value was assessed by comparing the global Chi-square values for each model. The Kaplan-Meier method was used for cumulative survival analysis with the log-rank test for assessing the statistical differences between the two groups according to the presence of each symptom. A two-sided p-value less than 0.05 was considered statistically significant.

## Results

### Demographic characteristics

The demographic characteristics of the patients according to symptom status are presented in Table 1. Among 128 patients, 80 (63%) patients were asymptomatic, and 47(37%) patients were symptomatic. Although there were no significant differences in age, body mass index, systolic blood pressure, or pulse pressure between the two groups, the symptomatic group had a higher proportion of females, hypertension, and use of diuretics than did the asymptomatic group. The major cause of symptoms in the symptomatic patients was dyspnea (63.8%), but New York Heart Association (NYHA) class III or IV dyspnea was rare (4.3%).

**Table 1:** Baseline clinical characteristics.

	Asymptomatic (n = 80)	Symptomatic (n = 47)	p-value
Age, years	57±14	59±12	0.358
Female	19(23.8)	23(48.9)	0.006
Body mass index, kg/m^2^	23.3±4.6	24.1±3.0	0.253
Systolic BP, mmHg	123.9±14.7	128.3±18.2	0.143
Diastolic BP, mmHg	75.6±9.7	81.3±12.4	0.005
Pulse pressure, mmHg	48.3±12.1	47.0±12.5	0.566
Hemoglobin, g/dl	13.5±1.9	12.6±1.93	0.184
Creatinine, mg/dl	0.95±0.32	1.02±0.59	0.413
EGFR, ml/min/1.73m^2^	80.7±22.6	78.2±19.8	0.319
Co-morbidities			
Hypertension	22 (27.5)	24 (51.1)	0.012
Diabetes mellitus	10 (12.5)	9 (19.1)	0.316
Dyslipidemia	8 (10.0)	10 (21.3)	0.113
Atrial fibrillation	4(5.0)	4 (8.5)	0.467
Chronic kidney disease	6(7.5)	6(12.8)	0.358
Medications			
Diuretics	13(16.3)	17 (36.2)	0.017
β-blocker	8 (10.0)	11(23.4)	0.069
CCB	12(15.0)	9 (19.1)	0.623
ACEi/ARB	37(46.3)	22 (46.8)	1.000
Symptoms			
Chest pain, or discomfort	0	15(31.9)	
Dyspnea			
NHYA Class (1/2/3/4)	80(100)/0/0/0	15(31.9)/30(63.8)/2(4.3)/0	
Pre-syncope, or syncope	0	4(8.5)	

Values are mean (±SD), number of subjects (%). ACEi, angiotensin-converting enzyme inhibitor; ARB, angiotensin receptor blocker; BP, blood pressure; CCB, calcium channel blocker; COPD, chronic obstructive pulmonary disease; EGFR, estimated glomelular filtration rate; NYHA, New York Heart Association. Chronic kidney disease was defined as creatinine clearance <60 ml/min.

### Echocardiographic characteristics

Table 2 shows the echocardiographic characteristics of the two groups. Of the patients with moderately dysfunctional BAVs, approximately one-third of patients displayed the A-P type of BAV, and there was no difference in the prevalence of the BAV phenotype between the two groups. The symptomatic group had a higher prevalence of moderate AS than did the asymptomatic group (72.3 vs. 43.8%, p = 0.003). Accordingly, a greater number of moderate AR patients were classified into the asymptomatic group.

**Table 2:** Echocardiographic characteristics.

	Asymptomatic (n = 80)	Symptomatic (n = 47)	p-value
BAV phenotype, n (%)			
A-P type	55 (68.8)	30 (63.8)	0.696
R-L type	25 (31.3)	17 (36.2)	
BAV dysfunction, n (%)			
Moderate AS	35 (43.8)	35 (72.3)	0.003
Moderate AR	36 (45.0)	7 (14.9)	<0.001
Moderate AS with AR	9 (11.3)	6 (12.8)	0.784
Aorta phenotype, n (%)			
Overall aortopathy	38 (47.5)	33(70.2)	0.016
Normal shape	42 (52.5)	14 (29.8)	0.016
Predominant sinus Valsalva	8 (10.0)	5 (10.6)	1.000
Predominant ascending aorta	30 (37.5)	28 (59.6)	0.018
Aorta dimension, mm			
Sinus of Valsalva	34.3 ± 5.1	34.5± 5.1	0.928
Sinotubularjunction	29.9 ± 4.7	30.4 ±4.4	0.533
Tubular portion of AA	38.6 ± 6.2	40.8 ± 6.1	0.057
Effective arterial elastance, mmHg/ml	1.5 ± 0.5	1.8 ± 0.4	0.003
Valvuloarterial impedance, mmHg/ml/m^2^	3.9±1.3	4.7±1.7	0.013
Echocardiography data			
Aortic valve area, cm^2^	1.25 ± 0.41	1.19 ± 0.38	0.521
Mean pressure gradient, mmHg	28 ± 7	30 ± 6	0.238
LVEDD, mm	51.3 ±5.3	49.0 ±4.6	0.019
LVESD, mm	33.1 ± 5.1	32.0 ±5.8	0.291
LAVI, ml/m^2^	24.6 ±9.1	29.9 ±11.4	0.006
RWT	0.39 ±0.06	0.42 ±0.07	0.036
LVMI, g/m^2^	111.5 ± 21.7	109.4 ± 25.6	0.612
LVEF, %	67.0 ± 5.9	67.2 ±6.4	0.883
E velocity, m/s	0.66±0.18	0.68±0.22	0.518
Deceleration time, msec	215 ±41	216 ±40	0.918
A velocity, m/s	0.71±0.19	0.76 ±0.22	0.231
e’ velocity, cm/s	6.5 ±2.2	5.5 ±1.7	0.003
A’ velocity, cm/s	8.6 ±1.7	8.0 ±1.7	0.105
S’ velocity, cm/s	6.7 ±1.4	5.9 ±1.5	0.003
E/e’	10.9 ±3.7	13.3 ± 4.9	0.002
RVSP, mmHg	25.6 ± 5.7	27.2 ± 6.3	0.179

Values are mean (±SD). LVEDD, left ventricular end-diastolic diameter; LVESD, left ventricular end-systolic diameter; IVSD, interventricualrseptal diameter; LVPWD, left ventricular posterior wall diameter; LAVI, left atrial voumeindex; RWT, relative wall thickness; LVMI, left ventricular mass index; LVEF, left ventricular ejection fraction; E, early diastolic mitral inflow; e’, early diastolic mitral annular; A, late diastolic mitral inflow; A’, late diastolic mitral annular; S’ peak systolic mitral annular; RVSP, Right ventricular systolic function.

Symptomatic patients showed a significantly higher prevalence of aortopathy than did asymptomatic patients (70.2 vs. 47.5%, p = 0.016). Among the three aortic phenotypes, there was a significantly higher prevalence of predominant AA in the symptomatic group than in the other group (59.6 vs. 37.5%, p = 0.018). Aortic diameters tended to be larger in patients with symptoms than in those without symptoms at the site of the tubular portion of the AA, with marginal statistical significance. In terms of noninvasively derived arterial stiffness, the effective arterial elastance was significantly higher in symptomatic patients than in those without symptoms (1.8±0.4 vs. 1.5±0.5 mmHg/ml, p = 0.003). Likewise, valvulo-arterial impedance calculated in AS patients (n = 85) was higher in the symptomatic group (4.7±1.7 vs. 3.9±1.3 mmHg/ml/m^2^, p = 0.013).

Symptomatic patients had a smaller LV end-diastolic dimension and higher relative wall thickness than did asymptomatic patients. There were no significant differences in LV mass index or LV ejection fraction (LVEF). The symptomatic group was found to possess more advanced LV diastolic dysfunction with a larger left atrium volume index, a lower e`velocity, and a higher E/e′ ratio than the asymptomatic group. Regarding the vascular-ventricular interaction, there were significant correlations between the structural and functional properties of the AA and LV diastolic indices. The AA diameters were well correlated with e′ velocity (r = -0.368, p<0.001) and E/e′ (r = 0.179, p = 0.044). The effective arterial elastance also revealed significant correlations with e′ velocity (r = -0.202, p = 0.023) and E/e′ (r = 0.182, p = 0.041).

### Determinants of symptomatic status

**Fig 2:**
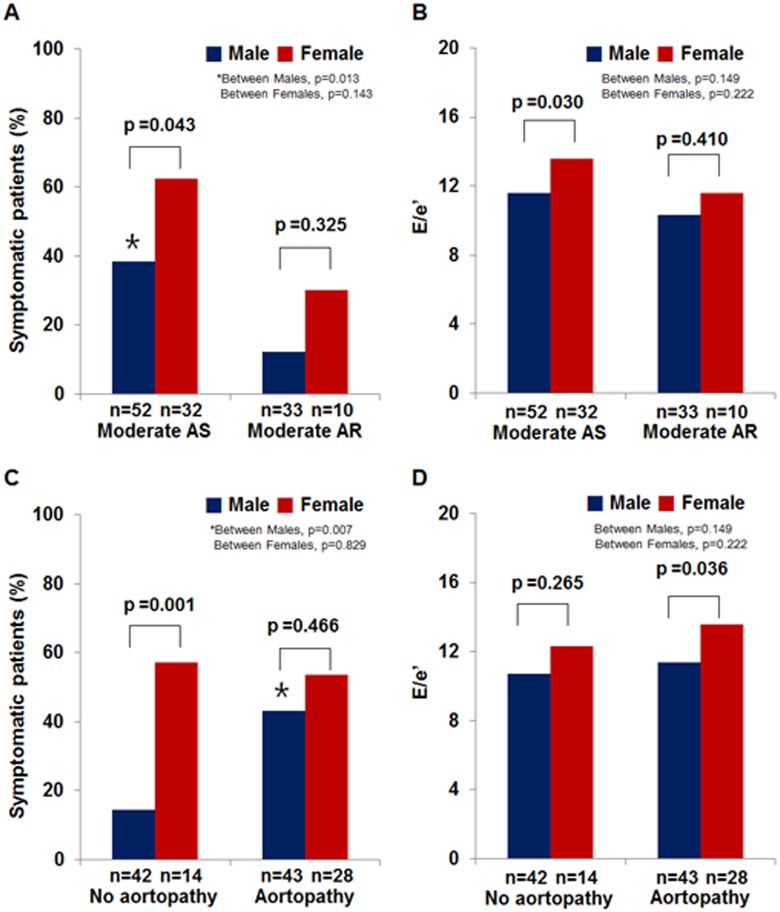
Presence of symptoms and E/e′ according to gender, BAV function, and aortopathy.

The percentages of patients with symptoms according to gender, moderate AS, or aortopathy are given in Fig 2. In patients with moderate AS, female patients displayed a higher rate of symptom presentation (62.5 vs. 37.5%, p = 0.043) and had a higher E/e′ (13.6±4.8 vs. 11.5±3.6, p = 0.030) than did male patients. In patients with aortopathy, female patients had a higher E/e′ (13.6±4.4 vs. 11.3±4.2, p = 0.036) than did male patients. However, although male patients overall had a lower prevalence of symptoms than did female patients, male patients with moderate AS or aortopathy had dramatic increases in symptom presentation (p = 0.013 and p = 0.007, respectively).

**Fig 3:**
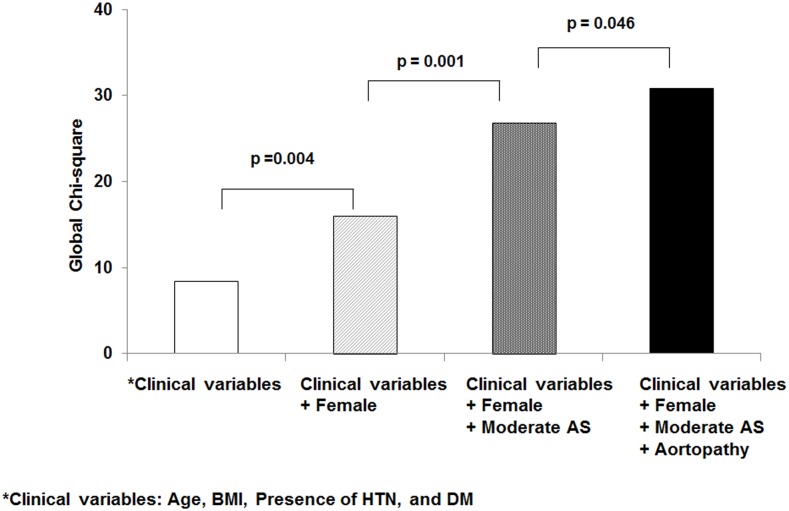
Incremental values for the presence of moderate AS and aortopathy in addition to clinical variables for the prediction of symptom status in patients with moderately dysfunctional BAVs.

Female gender, moderate AS, and the presence of aortopathy were predictive of symptomatic status in patients with moderately dysfunctional BAVs, even when taking into account a combination of clinical variables, including age, body mass index, and the presence of hypertension or diabetes mellitus (Fig 3).

When adjusting for confounding factors in the multivariate logistic regression analysis, female gender (odds ratio [OR] 2.84, 95% confidence interval [CI] 1.10–7.36, p = 0.031), hypertension (OR 3.07, 95% CI 1.20–7.82, p = 0.019), moderate AS (OR 5.33 5.78, 95% CI 1.99–16.83, p = 0.001), aortopathy (OR 2.76, 95% CI 1.07–7.10, p = 0.035), and E/e′ >15 (OR 3.82, 95% CI 1.03–11.19, p = 0.015) were associated with the symptomatic status of moderately dysfunctional BAV (Table 3).

**Table 3:** Determinants of symptom status in univariate and multivariate logistic regression analyses.

	Univariable		Multivariable	
	OR (95% CI)	p-value	OR (95% CI)	p-value
**Clinical characteristics**				
Age	1.01 (0.99–1.04)	0.355	0.98 (0.94–1.02)	0.260
Female	3.08 (1.43–6.64)	0.004	2.84 (1.10–7.36)	0.031
Body mass index	1.05 (0.96–1.16)	0.271	1.02 (0.92–1.13)	0.772
Hypertension	2.75 (1.30–5.85)	0.008	3.07 (1.20–7.82)	0.019
Diabetes mellitus	1.66 (0.62–4.31)	0.314	0.60 (0.16–2.31)	0.602
**Echocardiographic characteristics**				
BAV phenotype (A-P type)	1.25 (0.58–2.67)	0.570		
Moderate AS	4.68 (1.87–11.68)	0.001	5.78 (1.99–16.83)	0.001
Moderate AR	0.21 (0.09–0.53)	0.001		
AA dimension (mm)	1.06 (0.99–1.12)	0.060		
Presence of aortopathy	2.61 (1.21–5.59)	0.014	2.76 (1.07–7.10)	0.035
LVEF	1.01 (0.95–1.07)	0.882	0.95 (0.88–1.03)	0.215
LVMI	0.99 (0.98–1.01)	0.609	0.99(0.97–1.01)	0.483
LAVI	1.05 (1.01–1.09)	0.013		
E/e’	1.14 (1.04–1.25)	0.004		
e’ velocity	0.77 (0.63–0.94)	0.010		
E/e’>15	2.61 (1.21–5.59)	0.014	3.82 (1.03–11.19)	0.015

OR, odds ratio; CI, confidence interval; BAV, bicuspid aortic valve; A-P, anterior-posterior; AS, aortic stenosis; AR, aortic regurgitation; AA, ascending aorta; LVEF, left ventricular ejection fraction; LVMI, left ventricular mass index; LAVI, left atrial volume index; E, early diastolic mitral inflow; e’, early diastolic mitral annular

### Prognostic significance of symptomatic status

During a mean 41 ± 27 months of follow-up, adverse clinical events occurred in 31 (24.4%) patients (Table 4). In symptomatic patients compared to asymptomatic patients, AV surgery was performed more frequently (31.9 vs. 12.5%, p = 0.011) during the follow up period. AV surgery was underwent owing to symptomatic moderate AS (7 patients (14.9%)). Progression to severe AS was found in 8 patients (17.0%) in symptomatic group and 10 patients (12.5%) were in asymptomatic group (p = 0.481). The hospitalization for heart failure occurred more often in symptomatic patients (14.9% vs. 3.8%, p = 0.038). The LVEF of patients with hospitalization for heart failure was preserved (mean LVEF 65 ±5%).

**Table 4:** Clinical outcomes according to symptom status.

	Asymptomatic (n = 80)	Symptomatic (n = 47)	p-value
Composite outcome	14(17.5)	17(36.2)	0.031
Aortic valve surgery	10 (12.5)	15 (31.9)	0.011
Surgery for aortic valve only	3(3.8)	7 (14.9)	0.038
Surgery for aortic valve and aortic root	7 (8.8)	8 (17.0)	0.254
Hospitalization for heart failure	3 (3.8)	7 (14.9)	0.038
All-cause mortality	4 (5.0)	2 (4.3)	1.000

**Fig 4:**
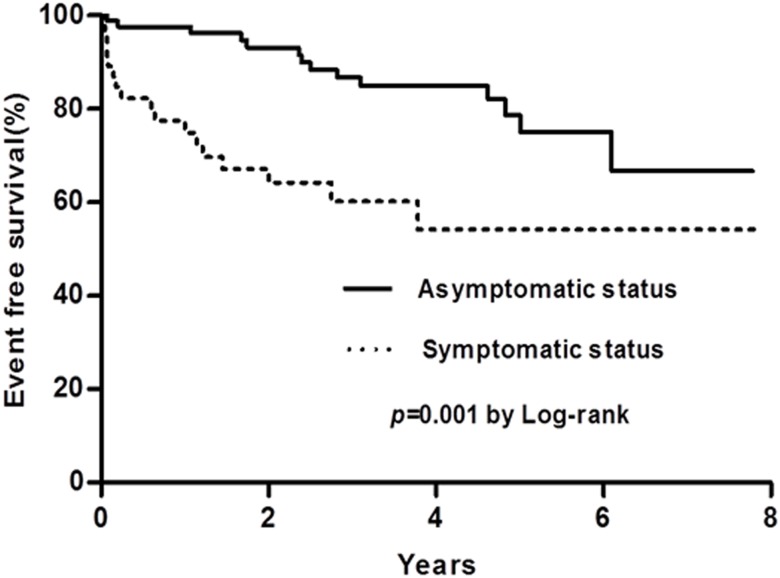
Kaplan-Meier survival curves showing a poorer prognosis of patients with symptoms compared to those without.

There was no significant difference in all-cause mortality between the two groups. The presence of symptoms was associated with reduced event-free survival during the follow-up period (54±9% vs. 68±10%, log-rank p = 0.001) (Fig 4).

## Discussion

The principal findings of the present study are that 1) symptoms are more prevalent in female patients, patients with hypertension, moderate AS, or aortopathy in association with consequent LV diastolic dysfunction in patients with moderately dysfunctional BAVs, and 2) more severe symptomatic status in patients with moderately dysfunctional BAVs was associated with poorer clinical outcomes. The results of our study demonstrate the clinical importance of BAV aortopathy in conjunction with other clinical factors that may affect increased LV afterload and consequent LV diastolic dysfunction. Therefore, risk stratification, careful follow-up, and identification of candidates for early AV surgery are necessary in patients with moderately dysfunctional BAVs.

Pathologic hypertrophy can lead to coronary microvascular dysfunction despite angiographically unobstructed coronary arteries [23]. When ventricular dilatation becomes detectable, pathological alterations such as reduced coronary blood flow per myocardial mass have already occurred [24]. A recent study demonstrated that coronary microvascular dysfunction is a main contributing factor to the development of chest pain in patients with severe AS without obstructive coronary artery disease [25]. Severe AS and LV remodelling reduce myocardial blood flow in the subendocardium, and reduction of flow is related to increased apoptosis of cardiomyoctes, which leads to heart failure [26]. Although coronary microvascular dysfunction has not been well established for explaining chest pain in moderate AS as much as in severe AS, it can exist in patients with moderate AS combined with increased LV afterload and consequent LV diastolic dysfunction.

The importance of heart failure with preserved ejection fraction is increasingly being recognised [27,28]. Patients with these conditions most likely develop heart failure owing to LV diastolic dysfunction. They tend to be older, to be female, and to have a history of hypertension [27,28]. Central aortic stiffness and the ventricular response to elevated LV afterload are highly linked to the pathogenesis of heart failure with preserved ejection fraction [21,29,30]. If there is AV disease in subjects who are vulnerable to heart failure, symptoms may occur more easily because of further pressure and volume overload in the LV. Moreover, if subjects with AV disease have dilatation of the proximal aorta and consequent central aortic stiffness, symptoms related to heart failure or AV disease can be further aggravated. In this study, the complaint symptoms of the 37% of patients with moderately dysfunctional BAVs were related to heart failure or AV disease. Symptomatic patients included more female patients and those with a higher prevalence of hypertension. Our results are in accordance with the generally proven risk factors for heart failure with preserved ejection fraction [27,28]. Symptomatic patients in this study possessed significantly elevated effective arterial elastance and valvulo-arterial impedance than did asymptomatic patients. Therefore, vascular stiffening combined with elevated systolic loads from AV disease, especially in moderate AS, may contribute to the clinical features of patients with moderately dysfunctional BAVs. Furthermore, symptomatic patients showed worse clinical outcomes than did asymptomatic patients. Even though the higher prevalence of symptomatic patients was probably influenced by the characteristics of the study population enrolled in a single tertiary care center, the present data showed a prognostic difference according to symptom status.

Several possible mechanisms can be suggested regarding the gender difference in symptom status in patients with moderately dysfunctional BAVs. In a previous study of 408 consecutive patients with isolated severe AS undergoing AV replacement, women were more symptomatic than men, but the affected women were also older and had smaller valve areas and higher mean pressure gradients than did the men [31]. Female patients were more symptomatic than male patients (23/42, 54.8% vs. 24/85, 28.2%, p = 0.006), even though the indexed AV area (0.73±0.24 vs. 0.73±0.22 cm^2^/m^2^, p = 0.936) and mean pressure gradient across the AV (28.4±6.9 vs. 29.9±5.8 mmHg, p = 0.311) did not differ by gender in the present study population. Female patients with moderately dysfunctional BAVs displayed more impaired LV diastolic functional parameters, including e′ velocity (5.6±1.9 vs. 6.5±2.2 cm/s, p = 0.026) and E/e′ (13.2±4.6 vs. 11.1±4.1, p = 0.011), compared to male patients. Our results are consistent with those of a few previous studies that demonstrated the importance of diastolic dysfunction on symptom status in severe AS, although the previous studies did not show gender-specific differences [8,32].

Previous studies reported that LV longitudinal relaxation was significantly impaired and estimated LV filling pressure was elevated in BAV subjects without significant valvular dysfunction [1,3,4]. There is a noticeable increase in central aortic stiffness in BAV subjects compared with TAV controls [2]. Moreover, independent correlations between the parameters of LV diastolic function and the indices of aortic mechanical function have been established in subjects with BAV [3]. Consistently, the present study demonstrated good correlations between the structural and functional properties of the AA and LV diastolic indices. We believe that valvular, arterial, and ventricular interplay is more important in patients with moderately dysfunctional BAVs than in subjects with normally functioning BAVs because either diastolic dysfunction or aortic stiffness may result in substantial clinical events.

## Limitations

There are several limitations to this study. First, the present study is a retrospective analysis; thus, an assessment of symptoms is dependent upon the accuracy of the medical records. Symptomatic patients were evaluated according to their NYHA class. We could not exclude patients who had occult coronary artery diseases, if those did not undergo evaluation of the coronary arteries. There were no objective tests for assessing exercise capacity in the present study. However, the mean age of the study population was 58±13 years-old; therefore, we suspect that the number of patients who were asymptomatic because they avoided physical activities would not represent a large portion of our patients. Second, this study was conducted in a tertiary care center, which raises the possibility that the study population may have a higher prevalence of co-morbidities than the general population. However, the prevalence of hypertension, the most important co-morbid condition influencing the results, was not high (36.2%). In addition, patients with coronary artery disease or specific co-morbidities influencing clinical outcomes were excluded. Third, this study included moderate AS with AR patients. Mixed aortic valve disease is found to be associated with a high rate of adverse events in patients with tricuspid aortic valves [33]. However, the prevalence of moderate AS with AR was not significantly different between the groups (11.3% vs. 12.8%, p = 0.784). Fourth, classification of aortopathy and evaluation of mechanical function only depended on the results of transthoracic echocardiography, although recent studies suggest that multidetector computed tomography or magnetic resonance imaging allows for an appropriate assessment of the extent of aortopathy and functional alteration of the aorta [34].

## Conclusions

In patients with moderately dysfunctional BAVs, symptom status is independently associated with female gender; the presence of hypertension, moderate AS, or aortopathy; and consequent LV diastolic dysfunction. Moreover, the presence of symptoms in moderately dysfunctional BAV patients is associated with worse clinical outcomes.

## Supporting Information

S1 File Minimal data set. (XLSX)
